# The Effects of Resistance Training on Sport-Specific Performance of Elite Athletes: A Systematic Review with Meta-Analysis

**DOI:** 10.5114/jhk/185877

**Published:** 2024-04-15

**Authors:** Hubert Makaruk, Marcin Starzak, Piotr Tarkowski, Jerzy Sadowski, Jason Winchester

**Affiliations:** 1 Department of Athletics, Faculty of Physical Education and Health, Józef Piłsudski University of Physical Education in Warsaw, Biała Podlaska, Poland.; 2 Faculty of Physical Education and Health, Józef Piłsudski University of Physical Education in Warsaw, Warsaw, Poland.; 3 Department of Sports and Training Science, Faculty of Physical Education and Health, Józef Piłsudski University of Physical Education in Warsaw, Biała Podlaska, Poland.; 4 Division of Health Sciences & Human Performance, Concordia University Chicago, Chicago, USA.

**Keywords:** training, strength exercise, strength and conditioning, plyometrics, sport-specific outcomes

## Abstract

This systematic review examines the influence of resistance training (RT) on the performance outcomes of elite athletes. Adhering to PRISMA guidelines, a comprehensive search across PubMed, Scopus, SPORTDiscus, and Web of Science databases was conducted, considering studies up to November 19, 2023. The inclusion criteria were elite athletes involved in high-level competitions. Studies were categorized by the competitive level among elite athletes, athlete's sex, performance outcomes, and a training modality with subgroup analyses based on these factors. Thirty-five studies involving 777 elite athletes were included. The results of the meta-analysis revealed a large and significant overall effect of RT on sport-specific performance (standardized mean difference, SMD = 1.16, 95% CI: 0.65, 1.66), with substantial heterogeneity (I^2^ = 84%). Subgroup analyses revealed differential effects based on the competitive level, the type of sport-specific outcomes, and sex. National elite athletes showed more pronounced (large SMD) benefits from RT compared to international elite athletes (small SMD). Global outcomes revealed a medium but non-significant (p > 0.05) SMD, while local outcomes showed a large SMD. Notably, female athletes exhibited a large SMD, though not reaching statistical significance (p > 0.05), probably due to limited study participants. No significant (p > 0.05) differences were found between heavy and light load RT. Resistance training is effective in improving sport-specific performance in elite athletes, with its effectiveness modulated by the competitive level, the type of the performance outcome, and athlete's sex. The findings underscore the need for personalized RT regimens and further research, particularly in female elite athletes, as well as advanced RT methods for international elite athletes.

## Introduction

Sport-specific performance is a complex, multi-dimensional concept crucial for success in competitive sports (Ford et al., 2011; Johnston et al., 2018). It is characterized by a unique blend of physical, technical, tactical, and psychological elements, each carefully tailored to the specific demands of a particular sport. This performance extends beyond basic physical attributes like strength, power, endurance, speed, agility, and flexibility (Tucker and Collins, 2012). It also includes mastering specialized movement patterns that are essential for effectively executing tasks unique to a sport (Elferink-Gemser et al., 2007). For example, the performance of a kick in soccer (Katis et al., 2013) or a serve in tennis (Etnyre, 1998) requires not just force but also accuracy, control, and decision-making. It is important to note that researching sport-specific performance entails significant challenges, primarily due to the intricacies involved in identifying key aspects or predictors of success within competitive sports. Direct evidence establishing predictors of competition success is scarce. The methodological constraints are particularly pronounced in dynamic and multifaceted sports disciplines, where capturing the essence of sport-specific performance is both critical and challenging (Chaabene et al., 2018). These challenges can be magnified in team sports where, outside of the attributes that a given athlete may possess or even technical and tactical components, dynamics of communication and interpersonal relationships can influence competitive outcomes. Despite these difficulties, in some systematic reviews, researchers have attempted to identify and evaluate sport-specific outcomes (Chaabene et al., 2018; Saeterbakken et al., 2022; Thiele et al., 2020).

Elite athletes compete at the highest levels of national and international sports. Their development in sports performance goes beyond innate talent to include unique training methods designed specifically for their advanced needs, which often differ from those of non-elite athletes (Lorenz et al., 2013). Defining the criteria to classify 'elite' athletes has been a challenging task for researchers, which has resulted in confusion and inconsistency in the literature. Although some attempts have been made to clarify the issue, as seen in Swann et al. (2015), there remains a considerable lack of consensus among researchers regarding this matter. Because of this, the term 'elite' in sports research spans a wide range, from athletes with only few years of training experience, to academy or university competitors, top-ranked participants, skilled national or international competitors, and extends to professionals, semi-professionals, world-class athletes, medalists, Olympians, and even Olympic or world champions (Williams et al., 2017). This absence of a universally accepted standard for what makes an athlete ‘elite’ presents a challenge to the validity of research focusing on high-level sports performance and training methods tailored to these athletes. It complicates the process of drawing reliable conclusions about the unique training and performance characteristics that distinguish elite athletes from their non-elite counterparts. In this work, we address this challenge by categorizing elite athletes as those competing at the highest international or national levels in their respective sports.

Resistance training (RT) is widely acknowledged as a vital component in enhancing athletic performance, significantly influencing an athlete's sport-specific abilities and skills (Abdi et al., 2019; Loturco et al., 2024; Spieszny et al., 2022; Thiele et al., 2020b). Its diverse nature allows for a broad spectrum of benefits, adaptable to various athletic needs and goals (Harries et al., 2012; Loturco et al., 2023a; Saeterbakken et al., 2022). This training method encompasses a wide range of tools, including free weights, machines, resistance bands, plyometrics, resisted sprint training, core stability exercises, and bodyweight exercises (Loturco et al., 2023b; Morris et al., 2022; Stone et al., 2000). The selection of tools and techniques can be specifically tailored to enhance particular muscle groups (Schoenfeld et al., 2019), improve overall strength, increase power or build endurance, depending on the athlete’s sport and individual requirements. However, this diversity also poses a challenge in identifying the most effective training regimen for each athlete, requiring a deep understanding of the sport's specific physical demands and the athlete’s personal performance goals (Harries et al., 2012; Thiele et al., 2020). For example, a basketball player may focus on plyometric exercises to improve explosive power and agility, vital for jumping and quick movements on the court. In contrast, a swimmer may utilize resistance bands to strengthen upper body muscles, crucial for effective stroke techniques in the water, and can deliberately avoid repetition ranges thought to be more effective at inducing muscle hypertrophy in an effort, to prevent an increase in their cross-sectional area.

In this systematic review, we address a critical gap in the current understanding of how RT specifically benefits elite athletes. Existing research predominantly focuses on the general physical advantages of RT, such as enhanced muscle mass and overall strength (Naclerio et al., 2013; Ratel, 2011; Zouita et al., 2023). However, when sport-specific performance is examined, participants are often not elite athletes (Harries et al., 2012), or studies combine data from elite athletes with those of sub-elite and recreational athletes, leading to less distinct findings (Thiele et al., 2020). This situation results in a lack of clarity on how adaptations derived from RT translate into enhanced sport-specific performance, particularly at the elite level. Our review aimed to synthesize available evidence to discern the specific impact of various RT methods on sport-specific skills and performance of elite athletes. The objective was twofold: firstly, to systematically analyze and integrate findings from existing studies, thereby establishing a clear understanding of the role of RT in elite sports performance. Secondly, the review sought to provide evidence-based recommendations for optimizing RT protocols for elite athletes, identifying strategies that would effectively enhance athletic performance. Such a focused approach is crucial for advancing the field of sports science and guiding coaches toward evidence-based practices in the development of elite-level athletes.

## Methods

### Search Strategy

This systematic review was conducted following the PRISMA guidelines (Moher et al., 2009). Given that the study did not entail direct involvement of human subjects, the institutional review board approval was not required. An extensive search was conducted across four electronic databases (PubMed, Scopus, SPORTDiscus, and Web of Science) using specific search terms combined with Boolean operators, as detailed in Table 1. This search included all publications available up to November 19, 2023, and was not limited by language.

**Table 1:** Searched terms used to identify potential studies.

Concept	Searched term
Independent variable (key issue)	"strength training" OR "weight training" OR "resistance training" OR "power training" OR "eccentric training" OR "isometric training" OR "strength exercise*" OR "weight exercise*" OR "resistance exercise*" OR "power exercise*" OR "eccentric exercise*" OR "isokinetic exercise*" OR "isometric exercise*" OR "heavy load*" OR "hypertrophy" OR "bodybuilding" OR "plyometric*" OR "Olympic lift*" OR "muscular endurance" OR "crossfit" OR "calisthenics" OR "free weight*" OR "machine exercise*" OR "machine weight*" OR "elastic bands" OR "weight vest" OR "weights belts" OR "core resistance" OR "core training" OR "trunk training" OR "medicine ball*" OR "kettlebell*" OR "resisted speed" OR "resisted sprint*" OR "resisted run*" OR "sled towing" OR "resisted sled" OR "uphill run*" OR "flywheel training" OR "flywheel resistance" OR "eccentric overload training" OR "isoinertial training" OR "Velocity Based Resistance Training" OR "Velocity-Based Strength Training" OR "VBT"
Dependent variable (sport skills as a result of learning)	"1 RM" OR "1RM" OR "rep* max*" OR "max* strength" OR "max* strength" OR "squat" OR "clean and jerk" OR "power clean" OR "snatch" OR "deadlift" OR "bench press" OR "leg press" OR "strength performance" OR "strength outcome*" OR "sprint" OR "speed run*" OR "run* time" OR "run* speed" OR "run* performance" OR "endurance run*" OR "run* endurance" OR "distance run*" OR "long distance run*" OR "run* economy" OR "run* distance" OR "run* outcome*" OR "sprint time" OR "1 repetition maximum" OR "sport-specific performance" OR "agility" OR "change of direction" OR "COD"
Population	"elite" OR "world-class athletes" OR "world-class players" OR "professional athletes" OR "highly trained" OR "highly skilled" OR "well-trained" OR "top athletes" OR "top-ranking athletes" OR "national team" OR "international level"
	#1 AND #2 AND #3

### Study Selection and Data Extraction

During the study selection and data extraction phase, titles, abstracts, and full texts were systematically evaluated by two independent reviewers (H.M., M.S.) according to the PICO criteria outlined in Table 2. The EndNote X9.3.3 software (Clarivate Analytics) was used to facilitate the removal of duplicate articles, which was complemented by a manual check to ensure thorough de-duplication. Any discrepancies encountered in the selection process were resolved through discussion until a consensus was reached.

**Table 2:** Selection criteria.

Category	Inclusion criteria	Exclusion criteria
Population	Elite female and male athletes who are international or national top-level athletes (succeeded in national competitions) or athletes in highly competitive leagues (e.g., NCAA Division 1)	Athletes competing at regional, state, or provincial levels, masters level competitors (> 35 years), the sample size includes both elite and non-elite (e.g., novice or recreational) athletes, athletes from countries with low sports competitiveness, disabled athletes
Intervention	Training programs lasting a minimum of 4 weeks or comprising at least 10 sessions; resistance training interventions including free weights, machine weights, isokinetic devices, elastic bands, resisted running, and plyometrics	Resistance training combined with supplementary aids (e.g., nutritional, pharmacological, physiological, or psychological)
Comparator	Control group consisting of elite athletes	Absence of a control or an active control group, athletes from sports different from the intervention group
Outcome	At least one measure of sport-specific performance	Lack of pre- and post-intervention measures, outcomes differing significantly from at least top national level
Study design	Between-subjects design	Systematic reviews, observational studies, case studies

Each study was coded for the following data: authors, sport, a competitive level (divided into categories: international level, national top-level), an intervention or a control group (resistance methods, e.g., strength training, power training, plyometrics, sprint resistance, trunk muscle training), the number of participants, sex, age, duration and frequency of intervention, the type of the performance test, outcomes (divided into categories: global, e.g., throwing velocity and local, e.g., running time). According to Thiele et al. (2020), multiple outcomes were ranked based on their significance for sport-specific performance, and the variable with the highest ranking was included in the subsequent analysis. Additionally, the types of resistance interventions were divided into two distinct categories: heavy and light (or power) load RT. Heavy RT typically involved lifting heavier weights (> 65% of one repetition maximum (1RM)) at a lower volume to increase muscle strength and hypertrophy, while light RT emphasized lifting lighter weights (< 60% of 1 RM) at a higher speed to enhance muscle power and speed of movement (Fisher et al., 2017). When study estimates were reported only graphically, data were extracted using ImageJ software version 1.53 (National Institutes of Health, USA, http://imagej.nih.gov).

### Study Quality

Two independent reviewers (M.S. and P.T.) assessed the risk of bias and methodological quality of eligible articles using the PEDro scale (Maher et al., 2003). Discrepancies in PEDro scores were resolved through consultation with a third, independent, PEDro-certified assessor (H.M.). Compliance with the PEDro scale's criteria was indicated by "yes" for criteria met and "no" for the unmet ones, following the guidelines provided by PEDro (see https://pedro.org.au/english/resources/pedro-scale/). The PEDro scale evaluates internal validity and the presence of statistically replicable information, using a scale from 0 (high risk of bias) to 10 (low risk of bias). A score of 6 or more represents the threshold for studies with a low risk of bias. Item 1 only pertains to external validity and is not included in the calculation of the overall PEDro score. In training intervention studies, blinding participants to the exercise program is impossible, and often, it is also not feasible to blind the investigators. Therefore, items 5 and 6 of the PEDro scale, which are related to blinding, were removed, reducing the maximum score to 8. Following the criteria of previous exercise intervention reviews (Kümmel et al., 2016; Saeterbakken et al., 2022; Starzak et al., 2024), studies were categorized as follows: 6–8 excellent quality, 5 good quality, 4 moderate quality, and 0–3 poor quality. Points were awarded only when a study explicitly met the criteria. Additionally, the PEDro scale was modified for this study to align more closely with the specific methodological aspects of the strength and conditioning field (Makaruk et al., 2022).

### Statistical Analysis

Between-subject standardized mean differences (SMDs) were calculated to determine the effects of RT on sport-specific performance, utilizing the formula: SMD = (mean1 − mean2) / s_pooled_, where 'mean1' was the mean pre/post-test value of the intervention group, 'mean2' was the mean pre/post-test value of the control group, and 's_pooled_' was the pooled standard deviation (Saeterbakken et al., 2022). The SMD was adjusted for sample size according to Hedges and Olkin (2014), using the factor (1 − (3 / (4N − 9))), with 'N' representing the total sample size. Adjusted SMD values, calculated as the difference between pre-test and post-test SMDs, were also determined (Durlak, 2009). A random effects model was applied to weigh each study according to its standard error and to aggregate the weighted mean adjusted SMDs. Positive SMD values were consistently reported when RT was found to have a favorable effect compared with controls. A *p* value of < 0.05 was considered indicative of statistical significance. SMD values were categorized as trivial (< 0.2), small (0.2 ≤ SMD < 0.5), medium (0.5 ≤ SMD < 0.8), or large (SMD ≥ 0.8) (Cohen, 2013).

The inclusion of studies in the meta-analysis required a minimum of two intervention groups. Subject-related moderator variables such as the performance level, the type of sport-specific outcomes, sex, and the type of the resistance method were examined through sub-group analyses. Meta-analyses were conducted using Review Manager (RevMan5.3, Copenhagen: The Nordic Cochrane Centre, The Cochrane Collaboration, 2014). The level of agreement between reviewers was assessed using Kappa correlation coefficients (Altman, 1990). These coefficients are generally interpreted as follows: 0.81–1.00 indicates very good agreement, 0.61–0.80 good, 0.41–0.60 moderate, 0.21–0.40 fair, and < 0.20 poor (Altman, 1990). The I^2^ statistic was utilized to assess the level of between-study heterogeneity (Higgins et al., 2003), with values of 25%, 50%, and 75% corresponding to low, moderate, and high heterogeneity, respectively. Values exceeding 75% were deemed highly heterogeneous. Additionally, the chi-square test was used to ascertain whether analysis results were due to chance, with significant results indicated by low *p*-values or high chi-square statistics relative to degrees of freedom (*df*).

## Results

### Study Selection

**Figure 1:**
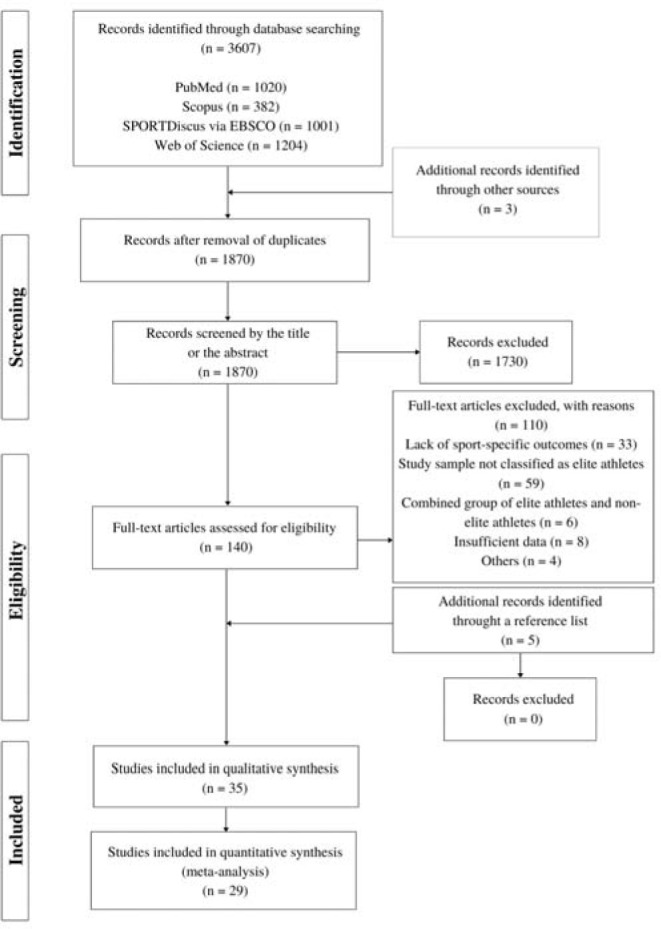
Flow chart of study selection for systematic review and meta-analysis.

A primary search of the electronic databases yielded 3607 studies. Additional three records were identified from other sources. After removing duplicates using Endnote software, and screening the titles and abstracts, 140 studies were selected for full-text revision. Five additional studies were found through a reference list search of eligible literature. Following all screening processes, 35 studies with 777 participants were included for qualitative analysis, and 29 studies were used for the meta-analysis. The flow of studies through the review process is reported in Figure 1.

### Methodological Quality

Table 3 provides a detailed analysis of the methodological quality of the studies using the PEDro scale. The median quality score was 4 points, with a range from 3 to 6 points, indicating moderate methodological quality. The agreement rate between the assessments performed by the two reviewers was classified as very good since the Kappa correlation coefficient was 0.94.

**Table 3:** PEDro methodological quality rating scores.

Study	Criterion											Total PEDro score
	1	2	3	4	5	6	7	8	9	10	11	
Aagaard et al. (2011)	0	1	0	1	0	0	0	0	0	1	1	4
Ben Brahim et al. (2021)	0	1	0	1	0	0	0	0	0	1	1	4
Blazevich and Jenkins (2002)	1	0	0	1	0	0	0	0	0	1	1	3
Born et al. (2020)	1	0	0	1	0	0	0	1	0	1	1	4
Büsch et al. (2015)	1	1	0	1	0	0	0	1	1	1	1	6
Carlsson et al. (2017)	0	1	0	1	0	0	0	0	0	1	1	4
Cherif et al. (2022)	1	0	0	1	0	0	0	0	0	1	1	3
Fernandez-Fernandez et al. (2013)	1	1	0	1	0	0	0	0	0	1	1	4
Hermassi et al. (2010)	1	1	0	1	0	0	0	0	0	1	1	4
Hermassi et al. (2015)	1	1	0	1	0	0	0	0	0	1	1	4
Hermassi et al. (2019)	1	1	0	1	0	0	1	1	0	1	1	6
Jones et al. (2018)	1	0	0	1	0	0	0	0	0	1	1	3
Karpiński et al. (2020)	1	1	0	1	0	0	0	0	0	1	1	4
Kristiansen et al. (2023)	1	1	0	1	0	0	0	0	0	1	1	4
Kristoffersen et al. (2019)	0	1	0	1	0	0	0	0	0	1	1	4
Losnegard et al. (2011)	1	0	0	1	0	0	0	1	0	1	1	4
McEvoy and Newton (1998)	1	0	0	1	0	0	0	0	0	1	1	3
Millet et al. (2002)	1	1	0	1	0	0	0	1	0	1	1	5
Newton and McEvoy (1994)	1	1	0	1	0	0	0	1	0	1	1	5
Newton et al. (1999)	1	1	0	1	0	0	0	1	0	1	1	5
Paavolainen et al. (1999)	0	0	0	1	0	0	0	1	0	1	1	4
Ramos Veliz et al. (2014)	1	1	0	1	0	0	0	0	0	1	1	4
Ramos Veliz et al. (2015)	1	1	0	1	0	0	0	1	0	1	1	5
Rønnestad et al. (2012)	1	0	0	1	0	0	0	1	0	1	1	4
Rønnestad et al. (2015)	1	1	0	1	0	0	0	1	0	1	1	5
Rønnestad et al. (2017)	1	1	0	1	0	0	0	1	0	1	1	5
Sabido et al. (2016)	0	1	0	1	0	0	0	0	0	1	1	4
Saez de Villarreal et al. (2014)	1	1	0	1	0	0	0	0	0	1	1	4
Saez de Villarreal et al. (2015a)	1	1	0	1	0	0	0	0	0	1	1	4
Saez de Villarreal et al. (2015b)	1	1	0	1	0	0	0	0	0	1	1	4
Saunders et al. (2006)	1	1	0	1	0	0	0	0	0	1	1	4
Sedano et al. (2009)	1	1	0	1	0	0	0	1	0	1	1	5
Sedano et al. (2013)	1	1	0	1	0	0	0	0	0	1	1	4
Therell et al. (2022)	1	0	0	1	0	0	0	0	0	1	1	3
Wang et al. (2022)	1	1	0	1	0	0	0	0	0	1	1	4

A detailed explanation of each item on the PEDro scale is available at https://pedro.org.au/english/resources/pedro-scale/.

None of the studies met the criterion of allocation concealment, with only one study explicitly stating whether an 'intention-to-treat' analysis was performed for the relevant outcomes. Moreover, except for one study, none of the studies included provided information on the criteria used for blinding methods.

### Study Characteristics

The pooled number of participants across the studies was 777, with a sample size ranging from 9 to 36 participants (Table 4). Twenty-five studies involved only male participants, two studies included only female athletes, and eight studies combined male and female participants. The participants' average age varied from 13.2 to 29.6 years. Out of the total, twenty-nine studies enrolled athletes who were adults (aged ≥ 18 years), whereas six studies involved youth athletes (aged ≤ 18 years). In twenty-six studies, athletes were categorized as national elites, while in nine studies, they were categorized as international elite athletes.

**Table 4a:** Study characteristics.

Study	Population		Intervention	Training period/ Frequency	Outcome
	Sport/ Competitive level	Sex, sample size (n), age (years ±SD)			
Aagaard et al. (2011)	Cycling/ International Elite	M, 19.5 (±0.8), EG: 7 CON: 7	EG: weighted strength training, CON: regular training	16 weeks, 2–3 sessions/week	45-min endurance test, capacity (W), EG1↑ > E2↑
Ben Brahim et al. (2021)	Soccer/ International Elite	M, 18.8 (±0.8), EG: 20 CON: 14	EG: resisted sprint training, CON: regular training	6 weeks, 2 sessions/week	Ball-shooting speed (km•h^−1^), EG↑ > CON↑
Blazevich and Jenkins (2002)	Sprinters/ National Elite	M, 19.0 (±1.4), EG1: 5 EG2: 4	EG1: strength training with high movement speeds, EG2: strength training with low movement speeds	7 weeks, 2 sessions/week	20-m acceleration (s), EG1↑ = EG2
Born et al. (2020)	Swimming/ International Elite	M&F, EG1: 10, 17.1 (±2.6), EG2: 11, 17.1 (±2.7)	EG1: heavy strength training, EG2: plyometric training	6 weeks, 2 sessions/week	25-m Freestyle swimming sprint (s), EG1 = EG2
Büsch et al. (2015)	Handball/ National Elite	M, EG1: 10, 16.7 (±0.6), EG2: 9, 17.4 (±0.9)	EG1: resistance training on stable ground, EG2: resistance training on unstable ground	10 weeks, 2 sessions/week	Figure eight run performance (s), EG1↑ = EG2↑
Carlsson et al. (2017)	Cross country skiing/ National Elite	M&F, EG1: 14, M: 18.4 (±1.0), F: 18.5 (±0.8), EG2: 19, M: 18.4 (±0.9), F: 17.7 (±0.9)	EG1: strength training, EG2: ski-ergometer training	6 weeks, 2 sessions/week	Maximal-speed test with the double-poling technique, maximal speed (km•h^−1^), EG1↑ = EG2↑
Cherif et al. (2022)	Handball/ National Elite	M, 22.1 (±3.0), EG: 11 CON: 11	EG: strength and power training, CON: regular training	12 weeks, 2 sessions/week	Throwing velocity (m·s^−1^), EG↑ = CON
Fernandez-Fernandez et al. (2013)	Tennis/ National Elite	M, EG: 15, 13.2 (±0.6) CON: 15, 13.2 (±0.5)	EG: resistance training, CON: regular training	6 weeks, 3 sessions/week	Serve velocity (km•h^−1^), EG↑ = CON
Hermassi et al. (2010)	Handball/ National Elite	M, EG1: 9, 20.0 (±0.5) EG2: 9, 20.1 (±0.6) CON: 8, 20.0 (±0.7)	EG1: heavy strength training, EG2: moderate strength training, CON: regular training	10 weeks, 2 sessions/week	Throwing velocity with run-up (m·s^−1^), EG1↑, EG2 > CON
Hermassi et al. (2015)	Handball/ National Elite	M, EG1: 10, 18.4 (±0.5) EG2: 12, 18.7 (±0.5) CON: 12, 18.5 (±0.5)	EG1: resistance training, EG2: throwing training, CON: regular training	8 weeks, 3 sessions/week	Throwing velocity with run-up (m·s^−1^), EG1↑ > CON; EG2 = CON

M: Men; W: Women; EG: Experimental group; CON: Control group; ↑: Indicates a significant increase

**Table 4b:** Study characteristics.

Study	Population		Intervention	Training period/ Frequency	Outcome
	Sport/ Competitive level	Sex, sample size (n), age (years ±SD)			
Hermassi et al. (2019)	Handball/ National Elite	M EG: 11, 20.3 (±0.5) CON: 11, 20.1 (±0.5)	EG: weighted strength training, CON: regular training	12 weeks, 2 sessions/week	Throwing velocity with run-up (m·s^−1^), EG↑ > CON
Jones et al. (2018)	Swimming/ International Elite	M&F, EG1: 4M, 2F, 19.4 (±1.1) EG2: 6M, 18.9 (±0.9)	EG1: weighted strength training, EG2: ballistic training	6 weeks, 3 sessions/week	Time to 5 m (s), EG1 = EG2
Karpiński et al. (2020)	Swimming/ National Elite	M, EG: 15, 20.2 (±1.2) CON: 15, 20.0 (±1.9)	EG: core muscle training, CON: regular training	6 weeks, 3 sessions/week	50-m swimming time (s), EG↑ = CON
Kristiansen et al. (2023)	Sprint Kayaking/ National Elite	M, 18.6 (±4.1), F, 17.0 (±1.4), EG1: 14 EG2: 12	EG1: strength training (improve), EG2: strength training (maintain)	6 weeks, 3 sessions/week	200-m kayak ergometer all-out test (s), EG1↑ = EG2
Kristoffersen et al. (2019)	Cycling/ National Elite	M&F, 29.6 (±0.6), EG1: 14 EG2: 6	EG1: resistance sprint training, EG2: heavy strength training	6 weeks, 2 sessions/week	5-min all-out cycling, average power output (W·kg^−1^), EG1↑ = EG2↑
Losnegard et al. (2011)	Cross-country skiing/ National Elite	M&F, EG: 9, M, 21.2 (±2.5), F, 21.3 (±5.1), CON: 10, M, 20.8 (±2.5), F, 22.6 (±2.4)	EG: heavy strength training, CON: regular training	12 weeks, 1–2 sessions/week	1.3-km skate-rollerski performance (VO_2 max_), EG = CON
McEvoy and Newton (1998)	Baseball/ International Elite	M, 24 (±4), EG: 9 CON: 9	EG: ballistic weight training, CON: regular training	10 weeks, 3 sessions/week/ every 2 weeks	Throwing speed (m·s^−1^), EG↑ > CON
Millet et al. (2002)	Triathlon/ International Elite	M, EG: 7, 24.3 (±5.2) CON: 8, 21.4 (±2.1)	EG: concurrent heavy weight training, endurance-strength training, CON: regular training	14 weeks, 2 sessions/week	3000-m running at V_Δ25%_, running economy (mL•kg^−1^•km^−1^), EG > CON
Newton and McEvoy (1994)	Baseball/ National Elite	M, 18.6 (±1.9), EG1: 8 EG2: 8 CON: 8	EG1: ballistic training, EG2: weighted strength training, CON: regular training	8 weeks, 2 sessions/week	Throwing speed (m·s^−1^), EG2↑ = EG1, CON
Newton et al. (1999)	Volleyball/ National Elite	M, 19 (±2), EG1: 8 EG2: 8	EG1: ballistic training, EG2: strength training	8 weeks, 2 sessions/week	Three-step vertical jump (cm), EG1↑ > EG2
Paavolainen et al. (1999)	Cross-country running/ National Elite	M, EG1: 10, 23 (3), EG2: 8, 24 (±5),	EG1: sport-specific explosive strength training with high training volume, CON: sport-specific explosive strength training with low training volume	9 weeks	5-km time trial (min), EG1↑ > CON

M: Men; W: Women; EG: Experimental group; CON: Control group; ↑: Indicates a significant increase

**Table 4c:** Study characteristics.

Study	Population		Intervention	Training period/ Frequency	Outcome
	Sport/ Competitive level	Sex, sample size (n), age (years ±SD)			
Ramos Veliz et al. (2014)	Water polo/ National Elite	M, 20.4 (±5.1), EG: 16 CON: 11	EG: strength training, CON: regular training	18 weeks, 2 sessions/week	Throwing velocity (m·s^−1^), EG↑ = CON
Ramos Veliz et al. (2015)	Water polo/ National Elite	F, 26.4 (±4.3), EG: 11 CON: 10	EG: lower body strength and power training, CON: regular training	16 weeks, 2 sessions/week	Throwing velocity (m·s^−1^), EG↑ = CON
Rønnestad et al. (2012)	Nordic Combined/ International Elite	M, EG: 8, 19 (±2) CON: 9, 20 (±3)	EG: heavy strength training, CON: regular training without heavy strength training	12 weeks, 2 sessions/week	7.5-km rollerski time-trial performance (s), EG = CON
Rønnestad et al. (2015)	Cycling/ International Elite	M, EG: 9, 19.1 (±1.7), CON: 7, 20.1 (±1.6)	EG: heavy strength training, CON: regular training	10/15 weeks, 1–2 sessions/week	Wingate test (VO_2max_), EG = CON
Rønnestad et al. (2017)	Cycling/ International Elite	M&F, EG: 10M, 2F, 19 (±2), CON: 6M, 2F, 20 (±2)	EG: heavy strength training, CON: regular training	10 weeks, 2 sessions/week	40-min all-out trial (VO_2max_) EG = CON
Sabido et al. (2016)	Handball/ National Elite	M, EG1: 12, 17.1 (±0.6), EG2: 11, 17.4 (±0.5), CON: 5, 17.0 (±0.6)	EG1: bench press throw exercise - known loads, EG2: bench press throw exercise - unknown loads, CON: regular training	4 weeks, 2 sessions/week	Jumping throw (km·h^−1^), EG2↑ = EG1, CON
Saez de Villarreal et al. (2014)	Water polo/ National Elite	M, EG1: 10, 19.7 (±5.4), EG2: 9, 18.5 (±2.3)	EG1: dry land resistance training, EG2: in water resistance training	6 weeks, 3 sessions/week	Throwing speed (km·h^−1^), EG1 = EG2
Saez de Villarreal et al. (2015a)	Water polo/ National Elite	M, 23.4 (±4.1), EG1: 10 EG2: 10 EG3: 10	EG1: strength and plyometric training, EG2: in water strength training, EG3: plyometric training	6 weeks, 3 sessions/week	Throwing speed (km·h^−1^), EG1↑ > EG3↑; EG1↑ = EG2↑
Saez de Villarreal et al. (2015b)	Soccer/ National Elite	M EG: 13, 15.3 (±0.3), CON: 13, 14.9 (±0.2)	EG: plyometric-sprint training, CON: regular training	9 weeks, 2 sessions/week	Ball-shooting speed (km·h^−1^), EG↑ > CON
Saunders et al. (2006)	Long and middle distance running/ National Elite	M, EG: 7, 23.4 (±3.2), CON: 8, 24.9 (±3.2)	EG: plyometric training, CON: regular training	9 weeks, 2–3 sessions/week	Treadmill running test, running economy at 18 km•h^−1^, (VO_2_; L•min^−1^), EG↑ > CON
Sedano et al. (2009)	Soccer/ National Elite	F, EG: 10, 22.8 (±2.1), CON: 10, 23.0 (±3.2)	EG: plyometric training, CON: regular training	12 weeks, 3 sessions/week	Kicking speed (km·h^−1^), EG↑ > CON

M: Men; W: Women; EG: Experimental group; CON: Control group; ↑: Indicates a significant increase

**Table 4d:** Study characteristics.

Study	Population		Intervention	Training period/ Frequency	Outcome
	Sport/ Competitive level	Sex, sample size (n), age (years ±SD)			
Sedano et al. (2013)	Soccer/ National Elite	M, EG: 11, 18.4 (±1.1), CON: 11, 18.2 (±0.9)	EG: plyometric training, CON: regular training	10 weeks, 3 sessions/week	Kicking speed (km·h^−1^), EG↑ > CON
Therell et al. (2022)	Cross-country skiing/ National Elite	M&F, EG1: 12, 18.1 (±0.6), EG2: 12, 18.0 (±1.5)	EG1: static core muscle training, EG2: dynamic core muscle training	9 weeks, 3 sessions/week	Roller ski treadmill test, energetic cost (J·kg^−1^·m^−1^), EG1 = EG2
Wang et al. (2022)	Volleyball/ National Elite	M, EG1: 6, 20.8 (±1.5), EG2: 6, 20.5 (±1.4), EG3: 6, 20.2 (±0.8)	EG1: high-load resistance training, EG2: low-load resistance training with blood flow restriction, EG3: high-load resistance training with blood flow restriction	8 weeks, 3 sessions/week	Three footed takeoff vertical jump (cm), EG3↑ > EG2; EG↑ = EG1

M: Men; W: Women; EG: Experimental group; CON: Control group; ↑: Indicates a significant increase

The duration of RT interventions varied from four to eight weeks, with training frequencies ranging from one to three sessions per week. The studies included in the analysis assessed sport-specific performance using various RT methods such as heavy strength training, ballistic training, plyometrics, concurrent strength and endurance training, core muscle training or different modalities of RT. These modalities included strength training on a stable and an unstable surface, dry-land and in-water strength training, as well as strength training with blood flow restriction, or combined methods of RT, including strength and plyometric training.

Twenty-three studies conducted comparisons between an intervention group that followed RT and an active control group that mostly followed a regular training routine in a specific sport, which was similar to the intervention group. Four of the twenty-three studies included two experimental groups compared to control groups. In addition, ten studies compared two different RT interventions without a control group, while two others compared three RT interventions exclusively.

### Main Analyses

**Figure 2:**
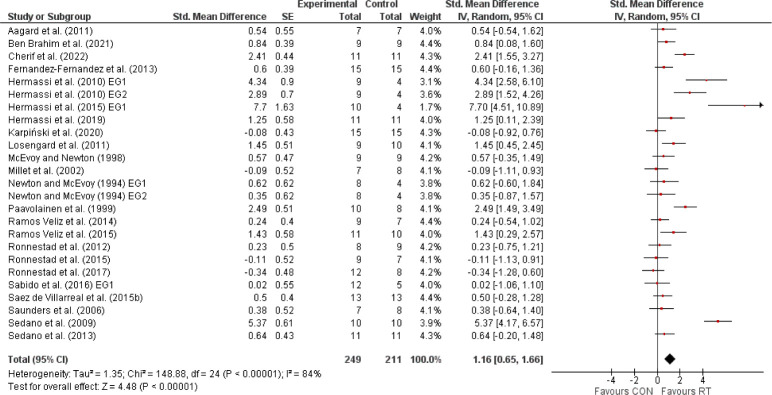
The effects of resistance training (RT) compared with a control group (CON) on sport-specific performance in elite athletes. *Experimental = experimental group, Control = control group, SMD = standardized mean difference, SE = standard error, Total = number of participants, CI = confidence interval, IV = inverse variance, Random = random effect model, df = degrees of freedom*.

**Figure 3:**
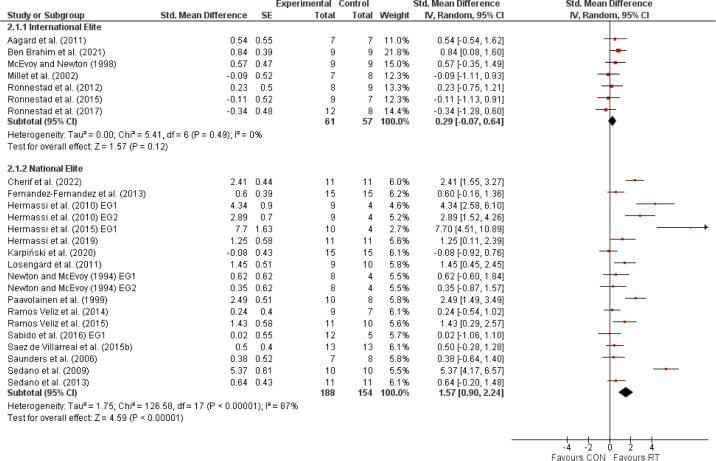
The effects of resistance training (RT) compared with a control group (CON) on sport-specific performance in elite athletes, delineated by the competitive level (international elite athletes, national elite athletes). *Experimental = experimental group, Control = control group, SMD = standardized mean difference, SE = standard error, Total = number of participants, CI = confidence interval, IV = inverse variance, Random = random effect model, df = degrees of freedom*.

**Figure 4:**
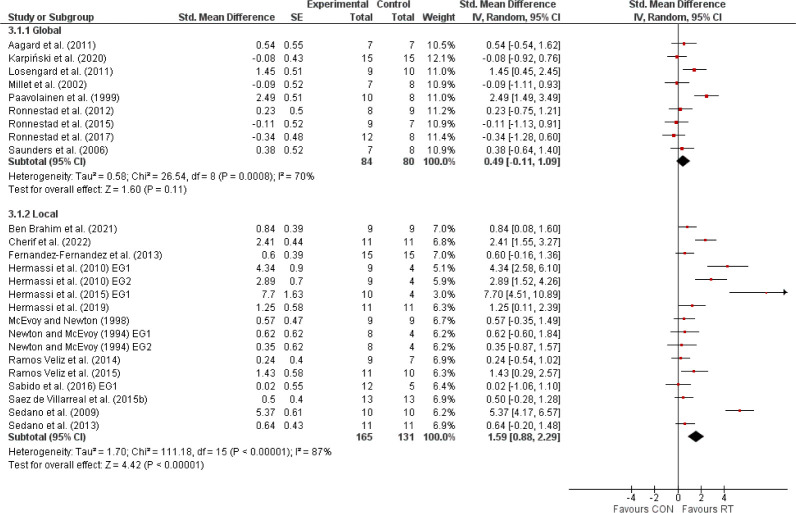
The effects of resistance training (RT) compared with a control group (CON) on sport-specific performance, categorized into global and local outcomes, in elite athletes. *Experimental = experimental group, Control = control group, SMD = standardized mean difference, SE = standard error, Total = number of participants, CI = confidence interval, IV = inverse variance, Random = random effect model, df = degrees of freedom*.

**Figure 5:**
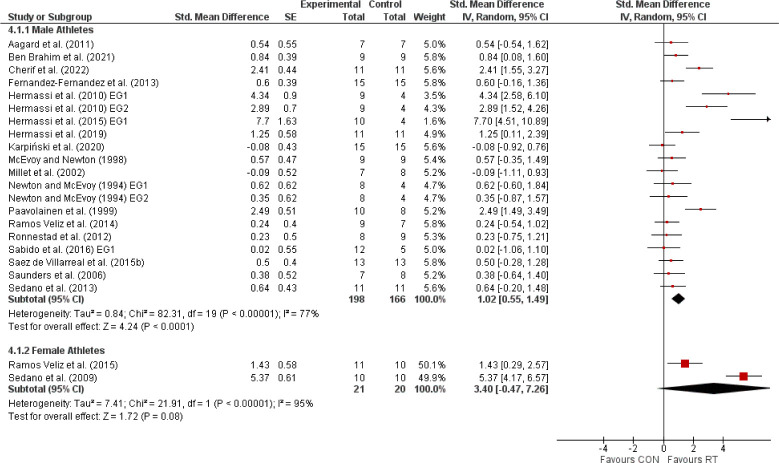
The effects of resistance training (RT) compared with a control group (CON) on sport-specific performance in elite athletes with a focus on sex-based differences. *Experimental = experimental group, Control = control group, SMD = standardized mean difference, SE = standard error, Total = number of participants, CI = confidence interval, IV = inverse variance, Random = random effect model, df = degrees of freedom*.

**Figure 6:**
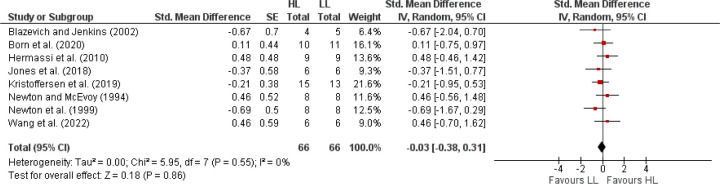
The effects of heavy loads resistance training (HL) compared with light loads resistance training (LL) on sport-specific performance in elite athletes. *Experimental = experimental group, Control = control group, SMD = standardized mean difference, SE = standard error, Total = number of participants, CI = confidence interval, IV = inverse variance, Random = random effect model, df = degrees of freedom*.

The primary meta-analysis, encompassing 23 studies with 460 elite athletes, demonstrated that RT significantly enhanced sport-specific performance, evidenced by a SMD of 1.16 (95% CI: 0.65, 1.66), indicative of a large effect size (Figure 2). Despite the noted heterogeneity (I^2^ = 84%), the overall positive effect was statistically significant (Z = 4.48, *p* < 0.00001). Subsequent analyses distinguished between international and national elite athletes (Figure 3). International elite athletes did not exhibit a statistically significant (*p* > 0.05) improvement in performance (SMD = 0.29; 95% CI: −0.07, 0.64; I^2^ = 0%), in contrast to national elite athletes who saw a larger, significant (*p* < 0.00001) benefit from RT (SMD = 1.57; 95% CI: 0.90, 2.24; I^2^ = 87%). A further breakdown revealed differential responses based on the type of the sport-specific outcome (Figure 4). Global outcomes yielded a medium but non-significant SMD of 0.49 (95% CI: −0.11, 1.09; *p* > 0.05; I^2^ = 70%), whereas local outcomes showed a more pronounced, significant effect (SMD = 1.59; 95% CI: 0.88, 2.29; *p* < 0.00001) with high heterogeneity (I^2^ = 87%). The sex-based analysis included 18 studies on male athletes and 2 on female athletes (Figure 5). The SMD for males was significant at 1.02 (95% CI: 0.55, 1.49; *p* < 0.00001; I^2^ = 77%), while females, despite a larger SMD of 3.40 (95% CI: −0.47, 7.26), did not reach statistical significance (*p* = 0.08), with very high heterogeneity (I^2^ = 95%). Lastly, the meta-analysis (Figure 6) comparing heavy versus light RT across eight studies found no significant (*p* > 0.05) differences in athletic performance outcomes (SMD = −0.03; 95% CI: −0.38, 0.31), and homogeneity in effects was observed (I^2^ = 0%).

## Discussion

The nature of each sport requires the development of specific skills to meet the demands and challenges of each sport, ensuring peak performance. Our analysis reveals that RT may play a pivotal role in sport-specific performance enhancement in elite athletes. The main conclusions of the conducted meta-analysis were that (i) RT produced large overall beneficial effects on sport-specific outcomes in elite athletes when compared with a control group of elite athletes, (ii) the effectiveness of RT in sport-specific performance was determined by a diverse range of factors, including the competitive level of athletes, the nature of outcomes, sex differences, and variations in RT methods, and (iii) there was a notable lack of randomized studies examining the influence of RT on sport-specific performance among international-level elite athletes, particularly those at Olympic or world champion levels, and a paucity of studies focusing on elite female athletes.

The findings of this systematic review revealed that RT, when used as a supplement to sport-specific training, offered large advantages (SMD greater than 1) for enhancing performance in various sports, from cross-country skiing (Losnegard et al., 2011), orienteering (Paavolainen et al., 1999) through handball (Hermassi et al., 2010) and soccer (Sedano et al., 2009) to water polo (Veliz et al., 2015). This enhancement demonstrates the versatile and impactful nature of RT in boosting sport-specific performance in elite athletes. A significant advantage was consistent across studies, as reflected by the high heterogeneity (I^2^ = 84%), suggesting that while RT is broadly effective, the degree of impact varies considerably among individuals. This variation could be due to differences in sport-specific demands, RT protocols, or individual athlete responses, underscoring the necessity for personalized RT regimens to optimize sport-specific performance enhancements in elite athletes. It is also possible that differences in prior experience with RT between athletes could have contributed to the variations noted above.

Interestingly, the subgroup analysis based on the level of competition revealed that RT had a more pronounced and significant effect on national elite athletes compared to their international counterparts. This may suggest that athletes at the national level likely have more room for physical improvement through RT compared to international athletes who may already be at or near their peak physical condition. Additionally, the observed low heterogeneity among international elite athletes might indicate more uniform training and performance standards at the highest sports level. In contrast, the diverse responses (high heterogeneity) within national elites could reflect a broader spectrum of training methods, physiological adaptations, and performance capacities. These insights point towards the need for a tailored approach in implementing RT, considering the athlete's current competitive level and specific needs (Loturco et al., 2023a). Thus, it becomes evident that a more specialized and advanced form of RT may be the key to unlocking further performance enhancements in international-level elite athletes. For instance, Zinke et al. (2018) found that in canoe sprint, where trunk muscles are crucial for both stabilizing the body in an unstable environment and generating propulsive forces, isokinetic training significantly enhanced athletic performance. Their study with world-class canoeists included a comprehensive 8-week isokinetic training program, emphasizing muscle hypertrophy and power. The findings revealed significant improvements in the peak torque of trunk rotators and established a strong correlation between the increased strength of concentric trunk rotators and peak paddle force, underscoring the efficacy of isokinetic training in boosting specific aspects of performance in elite canoe sprinters. Similarly, Schärer et al. (2019) explored the impact of a four-week eccentric-isokinetic training program on international and national top-level gymnasts, focusing on enhancing static strength elements in ring routines. This targeted approach yielded significant improvements in maximum strength for key elements such as the swallow and support scale. The success of this training regimen highlights the effectiveness of specific, tailored exercises for elite gymnasts. It is important to note that both the above-mentioned studies were single-group, non-randomized investigations. While they provided valuable insights into the effects of specialized training regimens on elite athletes, the lack of randomization and control groups in those studies limits the ability to generalize their findings. This highlights the need for more randomized controlled trials in this field to better understand the impacts of such training methods and to validate their effectiveness across a broader range of athletes. In addition, the smaller effect noted in international athletes does not necessarily suggest that RT was ineffective in that it is possible that cessation of current RT by such athletes could lead to a reduction in performance over time.

Typically, authors do not differentiate among various types of sport-specific outcomes. Both broad categories—such as running or swimming times—and more specific tasks like velocity in throwing or kicking are often analyzed together. To recognize this diversity and gain a more nuanced understanding of the impact of training interventions on sport-specific outcomes, we decided to separately examine the influence of RT on two distinct groups of outcomes: global and local. Global sport-specific outcomes, such as running, swimming, cycling, or rowing, engage multiple large muscle groups and often involve the entire body. These activities are typically considered whole-body movements and are used to assess overall performance in a given sport. Contrarily, local sport-specific outcomes focus on the performance of isolated muscle groups. This could be specific to the velocity of a kick in soccer, the accuracy of a throw in baseball or the power of a punch in boxing. They are crucial in sports where particular movements or actions are repetitive and critical for success. Meta-analyses of global and local sport-specific outcomes provide compelling evidence that RT has a differential impact on sport-specific performance, with a pronounced benefit for local sport tasks over global activities. On the one hand, this may reveal the multifaceted nature of global outcomes, indicating that they do not rely solely on one training method. On the other hand, it suggests that coaches have a tangible opportunity to effectively enhance technical elements of competitive tasks through RT. For instance, notable advancements in the velocity of ball-throwing or kicking have been observed in handball (Cherif et al., 2022; Hermassi et al., 2010; Hermassi et al., 2019), soccer (Ben Brahim et al., 2021; Campo et al., 2009) and water polo (Veliz et al., 2015). This evidence highlights the crucial need to understand how incorporating RT methods can specifically influence sport-related outcomes, thereby enabling a more effective and tailored approach to meet the distinct requirements of each sport.

An additional meta-analysis conducted as part of this study highlighted the impact of RT on sport-specific outcomes concerning sex differences. It is clear that RT benefits both male and female athletes, a finding that aligns with previous research (Kojić et al., 2021); however, the evidence suggests more substantial gains in male athletes. The observed positive trends in female athletes likely did not reach statistical significance, potentially due to the limited number of studies focused on elite female athletes (Campo et al., 2009; Veliz et al., 2015). This paucity of research on female athletes emphasizes the critical need for more comprehensive studies that investigate the effects of RT on sport-specific outcomes in elite female cohorts.

The findings of the final meta-analysis revealed no significant differences between heavy and light load RT, with notably low heterogeneity (I^2^ = 0%). These results imply that RT adaptations may align more closely with the unique performance requirements of their sports rather than the intensity of the load. This concept of specificity is particularly relevant in the context of team sports and multi-discipline events (Reilly et al., 2009), where performance requirements are diverse and require a holistic approach to training. Further, supporting the concept that RT adaptations are closely linked to sport-specific demands is the consistency observed across various RT studies in both individual and team sports used in this meta-analysis. This understanding may underscore the importance of a customized training approach, one that acknowledges and addresses the intricacies of each sport and athlete. It also highlights the need for training regimens that are not only physically demanding but also strategically aligned with the specific skills, tactics, and team dynamics essential for success in competitive sports.

The primary limitation of this study, which is also prevalent in RT literature, is the tendency to aggregate various types of sport-specific outcomes. This approach may obscure the distinct effects of RT on global versus local sport tasks, potentially masking the nuanced benefits of specific skills and highlighting the necessity for separate analyses to fully comprehend RT's impact on diverse performance metrics. This also highlights the need to identify sport-specific key performance indicators that are predictive of success, which can then serve as the outcome measures for future investigations. Future research should focus on the development of sport-specific resistance training protocols that account for the unique physiological and biomechanical demands of different sports, aiming to optimize individual athlete performance and reduce injury risk. Additionally, there is a need to explore the longitudinal effects of resistance training on athletes’ development, particularly how variations in training loads, frequency, and intensity over time influence sport-specific skills and performance outcomes. As noted earlier, there is a scarcity of research performed on women as compared to men looking into the magnitude of impact that resistance training has on sport performance. As such, future investigations should explore whether similar relationships exist between men and women and what are, if any, sex-specific differences. We also suggest that studies looking into potential thresholds of strength and power in relation to different levels of competition be performed. This may help solidify the understanding of how influential resistance training is for athletes at different performance levels, i.e., national versus international competition, and indicate potential implications of prioritization of resistance training in the overall training plan. Finally, the results of this review suggest that there exists a good deal of individuality in the magnitude of response to resistance training and the relevant relationship with sport performance. Future investigations may wish to explore this phenomenon in an effort to optimize outcomes for all athletes.

The systematic review and meta-analysis conducted herein underscore the significance of RT as supplementary to sport-specific training in enhancing performance of elite athletes. Resistance training has demonstrated large beneficial effects across a range of sports, indicating its efficacy in improving sport-specific performance, from endurance to high-power sports and from individual to team sports. However, the influence of RT is not uniform; it varies according to the athlete's competitive level, sex, and the specific demands of their sport. Notably, the impact of RT is more pronounced in national elite athletes than their international counterparts, suggesting that the latter may require more advanced and individualized training interventions (Loturco et al., 2024). Further meta-analysis revealed that RT had a more significant impact on local sports tasks compared to global activities, indicating its differential influence on sport-specific performance. The studies also identified a critical research gap regarding the effects of RT on female elite athletes, emphasizing an urgent need for further investigation. Overall, the findings advocate for the principle of specificity in RT programs, with a balanced integration of heavy and light loads tailored to the athlete's unique needs and the performance requirements of their sport. Finally, this review calls for more randomized controlled trials, especially among international-level elite and female athletes, to confirm the generalizability of these findings and to refine RT protocols for optimal sport-specific outcome enhancement.

## Conclusions

This study underscores the importance for coaches to design RT programs that are specifically tailored to the unique needs of each athlete, factoring in their sport, competitive level, and sex, to maximize sport-specific performance enhancements. The current body of literature suggests that at lower levels of competition, resistance training provides greater impact on sport performance, perhaps due to differing levels of conditioning at the international level. It also highlights the necessity of developing advanced and individualized RT interventions for international elite athletes and addressing the research gap between the sexes, ensuring that training protocols are optimized for all athletes.

## References

[ref1] Aagaard, P., Andersen, J. L., Bennekou, M., Larsson, B., Olesen, J. L., Crameri, R., Magnusson, P., & Kjær, M. (2011). Effects of resistance training on endurance capacity and muscle fiber composition in young top-level cyclists. Scandinavian Journal of Medicine & Science in Sports, 21(6), E298–E307. DOI: 10.1111/j.1600-0838.2010.01283.x 21362056

[ref2] Abdi, N., Hamedinia, M.R., Izanloo , Z., & Hedayatpour, N. (2019). The effect of linear and daily undulating periodized resistance training on the neuromuscular function and the maximal quadriceps strength. Balt J Health Phys Activ, 11, 45-53. 10.29359/BJHPA.11.1.05

[ref3] Altman, D. G. (1990). Practical statistics for medical research. CRC Press.

[ref4] Ben Brahim, M., Bougatfa, R., Makni, E., Gonzalez, P. P., Yasin, H., Tarwneh, R., Moalla, W., & Elloumi, M. (2021). Effects of combined strength and resisted sprint training on physical performance in U-19 elite soccer players. Journal of Strength and Conditioning Research, 35(12), 3432–3439. DOI: 10.1519/JSC.0000000000003829 33065708

[ref5] Blazevich, A. J., & Jenkins, D. G. (2002). Effect of the movement speed of resistance training exercise on sprint and strength performance in concurrently training elite junior sprinters. Journal of Orthopaedic & Sports Physical Therapy, 33(5), 290–291. 10.1080/026404102321011742 12477008

[ref6] Born, D.-P., Stöggl, T., Petrov, A., Burkhardt, D., Lüthy, F., & Romann, M. (2020). Analysis of freestyle swimming sprint start performance after maximal strength or vertical jump training in competitive female and male junior swimmers. Journal of Strength & Conditioning Research, 34(2), 323–331. DOI: 10.1519/JSC.0000000000003390 31985714

[ref7] Büsch, D., Pabst, J., Mühlbauer, T., Ehrhardt, P., & Granacher, U. (2015). Effects of plyometric training using unstable surfaces on jump and sprint performance in young sub-elite handball players. Sports Orthopaedics and Traumatology, 31(4), 299–308. 10.1016/j.orthtr.2015.07.007

[ref8] Carlsson, T., Wedholm, L., Nilsson, J., & Carlsson, M. (2017). The effects of strength training versus ski-ergometer training on double-poling capacity of elite junior cross-country skiers. European Journal of Applied Physiology, 117, 1523–1532. DOI 10.1007/s00421-017-3621-1 28597103 PMC5506237

[ref9] Chaabene, H., Negra, Y., Bouguezzi, R., Capranica, L., Franchini, E., Prieske, O., Hbacha, H., & Granacher, U. (2018). Tests for the assessment of sport-specific performance in Olympic combat sports: A systematic review with practical recommendations. Frontiers in Physiology, 9, 386. 10.3389/fphys.2018.00386 29692739 PMC5902544

[ref10] Cherif, M., Said, M. A., Ben Chaifa, M., & Kotb, A. A. H. (2022). Position-dependent morning-to-evening variability in physical performances in elite male handball players. Biological Rhythm Research, 53(10), 1496–1508. 10.1080/09291016.2021.1967574

[ref11] Cohen, J. (2013). *Statistical power analysis for the behavioral sciences*. Academic Press.

[ref12] Durlak, J. A. (2009). How to select, calculate, and interpret effect sizes. Journal of Pediatric Psychology, 34(9), 917–928. 10.1093/jpepsy/jsp004 19223279

[ref13] Fernandez-Fernandez, J., Ellenbecker, T., & Ulbricht, A. (2013). Effects of a 6-week junior tennis conditioning program on service velocity. Journal of Sports Science & Medicine, 12(2), 232–239. 24149801 PMC3761833

[ref14] Elferink-Gemser, M. T., Visscher, C., Lemmink, K. A., & Mulder, T. (2007). Multidimensional performance characteristics and standard of performance in talented youth field hockey players: A longitudinal study. Journal of Sports Sciences, 25(4), 481–489. 10.1080/02640410600719945 17365535

[ref15] Etnyre, B. R. (1998). Accuracy characteristics of throwing as a result of maximum force effort. Perceptual and Motor Skills, 86(3_suppl), 1211–1217. 10.2466/pms.1998.86.3c.1211 9700796

[ref16] Fisher, J., Steele, J., & Smith, D. (2017). High-and low-load resistance training: interpretation and practical application of current research findings. Sports Medicine, 47, 393–400. DOI 10.1007/s40279-016-0602-1 27480764

[ref17] Ford, P., De Ste Croix, M., Lloyd, R., Meyers, R., Moosavi, M., Oliver, J., Till, K., & Williams, C. (2011). The long-term athlete development model: Physiological evidence and application. Journal of Sports Sciences, 29(4), 389–402. 10.1080/02640414.2010.536849 21259156

[ref18] Harries, S. K., Lubans, D. R., & Callister, R. (2012). Resistance training to improve power and sports performance in adolescent athletes: a systematic review and meta-analysis. Journal of Science and Medicine in Sport, 15(6), 532–540. 10.1016/j.jsams.2012.02.005 22541990

[ref19] Hedges, L. V., & Olkin, I. (2014). Statistical methods for meta-analysis. Academic Press.

[ref20] Hermassi, S., Chelly, M. S., Fathloun, M., & Shephard, R. J. (2010). The effect of heavy-vs. moderate-load training on the development of strength, power, and throwing ball velocity in male handball players. Journal of Strength & Conditioning Research, 24(9), 2408–2418. DOI: 10.1519/JSC.0b013e3181e58d7c 20706155

[ref21] Hermassi, S., Van den Tillaar, R., Khlifa, R., Chelly, M. S., & Chamari, K. (2015). Comparison of in-season-specific resistance vs. a regular throwing training program on throwing velocity, anthropometry, and power performance in elite handball players. Journal of Strength & Conditioning Research, 29(8), 2105–2114. doi: 10.1519/jsc.0000000000000855 25627646

[ref22] Hermassi, S., Haddad, M., Laudner, K. G., & Schwesig, R. (2019). Comparison of a combined strength and handball-specific training vs. isolated strength training in handball players studying physical education. Sportverletzung Sportschaden, 33(03), 149–159. DOI: 10.1055/a-0919-7267 31419809

[ref23] Higgins, J. P., Thompson, S. G., Deeks, J. J., & Altman, D. G. (2003). Measuring inconsistency in meta-analyses. BMJ, 327(7414), 557–560. DOI: 10.1136/bmj.327.7414.557 12958120 PMC192859

[ref24] Johnston, K., Wattie, N., Schorer, J., & Baker, J. (2018). Talent identification in sport: a systematic review. Sports Medicine, 48, 97–109. 10.1007/s40279-017-0803-2 29082463

[ref25] Jones, J. V., Pyne, D. B., Haff, G. G., & Newton, R. U. (2018). Comparison of ballistic and strength training on swimming turn and dry-land leg extensor characteristics in elite swimmers. International Journal of Sports Science & Coaching, 13(2), 262–269. 10.1177/1747954117726017

[ref26] Karpiński, J., Rejdych, W., Brzozowska, D., Gołaś, A., Sadowski, W., Swinarew, A. S., Stachura, A., Gupta, S., & Stanula, A. (2020). The effects of a 6-week core exercises on swimming performance of national level swimmers. PLoS ONE, 15(8), e0227394. 10.1371/journal.pone.0227394 32866148 PMC7458297

[ref27] Katis, A., Giannadakis, E., Kannas, T., Amiridis, I., Kellis, E., & Lees, A. (2013). Mechanisms that influence accuracy of the soccer kick. Journal of Electromyography and Kinesiology, 23(1), 125–131. 10.1016/j.jelekin.2012.08.020 23021602

[ref28] Kojić, F., Mandić, D., & Ilić, V. (2021). Resistance training induces similar adaptations of upper and lower-body muscles between sexes. Scientific Reports, 11(1), 23449. 10.1038/s41598-021-02867-y 34873221 PMC8648816

[ref29] Kümmel, J., Kramer, A., Giboin, L.-S., & Gruber, M. (2016). Specificity of balance training in healthy individuals: a systematic review and meta-analysis. Sports Medicine, 46, 1261–1271. 10.1007/s40279-016-0515-z 26993132

[ref30] Kristiansen, M., Pedersen, A., Sandvej, G., Jorgensen, P., Jakobsen, J. V., de Zee, M., Hansen, E. A., & Klitgaard, K. K. (2023). Enhanced Maximal Upper-Body Strength Increases Performance in Sprint Kayaking. Journal of Strength and Conditioning Research, 37(4), E305–E312. 10.1519/JSC.0000000000004347 36731004

[ref31] Kristoffersen, M., Sandbakk, O., Ronnestad, B. R., & Gundersen, H. (2019). Comparison of Short-Sprint and Heavy Strength Training on Cycling Performance. Frontiers in Physiology, 10, 1132. 10.3389/fphys.2019.01132 31555153 PMC6724228

[ref32] Lorenz, D. S., Reiman, M. P., Lehecka, B., & Naylor, A. (2013). What performance characteristics determine elite versus nonelite athletes in the same sport? Sports Health, 5(6), 542–547. 10.1177/1941738113479763 24427430 PMC3806174

[ref33] Losnegard, T., Mikkelsen, K., Ronnestad, B. R., Hallén, J., Rud, B., & Raastad, T. (2011). The effect of heavy strength training on muscle mass and physical performance in elite cross country skiers. Scandinavian Journal of Medicine & Science in Sports, 21(3), 389–401. 10.1111/j.1600-0838.2009.01074.x 20136751

[ref34] Loturco, I., Zabaloy, S., Pereira, L., Moura, T., Mercer, V., Fernandes, V., ... & Bishop, C. (2024). Resistance training practices of Brazilian Olympic sprint and jump coaches: toward a deeper understanding of their choices and insights (part III). Journal of Human Kinetics, 90, 183–214. 10.5114/jhk/182888 38380293 PMC10875694

[ref35] Loturco, I., Freitas, T., Zabaloy, S., Pereira, L., Moura, T., Fernandes, V. … Bishop, C. (2023a). Speed training practices of Brazilian Olympic sprint and jump coaches: toward a deeper understanding of their choices and insights (part II). Journal of Human Kinetics, 89, 187–211. 10.5114/jhk/174071 38053953 PMC10694730

[ref36] Loturco, I., Haugen, T., Freitas, T. T., Bishop, C., Moura, T. B. M. A., Mercer, V. P. … Weldon, A. (2023b). Strength and conditioning practices of Brazilian Olympic sprint and jump coaches. Journal of Human Kinetics, 86, 175–194. 10.5114/jhk/15964 37181261 PMC10170547

[ref37] Maher, C. G., Sherrington, C., Herbert, R. D., Moseley, A. M., & Elkins, M. (2003). Reliability of the PEDro scale for rating quality of randomized controlled trials. Physical Therapy, 83(8), 713–721. 10.1093/ptj/83.8.713 12882612

[ref38] Makaruk, H., Starzak, M., Płaszewski, M., & Winchester, J. B. (2022). Internal Validity in Resistance Training Research: A Systematic Review. Journal of Sports Science & Medicine, 21(2), 308–331. doi: 10.52082/jssm.2022.308 35719235 PMC9157516

[ref39] McEvoy, K. P., & Newton, R. U. (1998). Baseball throwing speed and base running speed: The effects of ballistic resistance training. Journal of Strength & Conditioning Research, 12(4), 216–221.

[ref40] Moher, D., Liberati, A., Tetzlaff, J. & Altman, D.G. (2009). Preferred reporting items for systematic reviews and meta-analyses: the PRISMA statement. Annals of Internal Medicine, 151, 264–269. 10.1016/j.ijsu.2010.02.007 19622511

[ref41] Morris, S. J., Oliver, J. L., Pedley, J. S., Haff, G. G., & Lloyd, R. S. (2022). Comparison of weightlifting, traditional resistance training and plyometrics on strength, power and speed: a systematic review with meta-analysis. Sports Medicine, 52(7), 1533–1554. 10.1007/s40279-021-01627-2 35025093 PMC9213388

[ref42] Naclerio, F., Faigenbaum, A. D., Larumbe-Zabala, E., Perez-Bibao, T., Kang, J., Ratamess, N. A., & Triplett, N. T. (2013). Effects of different resistance training volumes on strength and power in team sport athletes. Journal of Strength & Conditioning Research, 27(7), 1832–1840. doi: 10.1519/jsc.0b013e3182736d10 23044934

[ref43] Newton, R. U., & McEvoy, K. I. (1994). Baseball throwing velocity: A comparison of medicine ball training and weight training. Journal of Strength & Conditioning Research, 8(3), 198–203.

[ref44] Newton, R. U., Kraemer, W. J., & Hakkinen, K. (1999). Effects of ballistic training on preseason preparation of elite volleyball players. Medicine & Science in Sports & Exercise, 31(2), 323–330. 10063823 10.1097/00005768-199902000-00017

[ref45] Paavolainen, L., Hakkinen, K., Hamalainen, I., Nummela, A., & Rusko, H. (1999). Explosive-strength training improves 5-km running time by improving running economy and muscle power. Journal of Applied Physiology, 86(5), 1527–1533. 10.1152/jappl.1999.86.5.1527 10233114

[ref46] Ramos Veliz, R. R., Requena, B., Suarez-Arrones, L., Newton, R. U., & De Villarreal, E. S. (2014). Effects of 18-week in-season heavy-resistance and power training on throwing velocity, strength, jumping, and maximal sprint swim performance of elite male water polo players. Journal of Strength & Conditioning Research, 28(4), 1007–1014. DOI: 10.1519/JSC.0000000000000240 24077370

[ref47] Ramos Veliz, R., Suarez-Arrones, L., Requena, B., Haff, G. G., Feito, J., & de Villarreal, E. S. (2015). Effects of in-competitive season power-oriented and heavy resistance lower-body training on performance of elite female water polo players. Journal of Strength & Conditioning Research, 29(2), 458–465. DOI: 10.1519/JSC.0000000000000643 25144134

[ref48] Ratel, S. (2011). High-intensity and resistance training and elite young athletes. Elite Young Athlete, 56, 84–96. 10.1159/000320635 21178368

[ref49] Reilly, T., Morris, T., & Whyte, G. (2009). The specificity of training prescription and physiological assessment: A review. Journal of Sports Sciences, 27(6), 575–589. 10.1080/02640410902729741 19340630

[ref50] Rønnestad, B. R., Kojedal, Ø., Losnegard, T., Kvamme, B., & Raastad, T. (2012). Effect of heavy strength training on muscle thickness, strength, jump performance, and endurance performance in well-trained Nordic Combined athletes. European Journal of Applied Physiology, 112, 2341–2352. 10.1007/s00421-011-2204-9 22038144

[ref51] Rønnestad, B. R., Hansen, J., Hollan, I., & Ellefsen, S. (2015). Strength training improves performance and pedaling characteristics in elite cyclists. Scandinavian Journal of Medicine & Science in Sports, 25(1), e89–e98. 10.1111/sms.12257 24862305

[ref52] Rønnestad, B. R., Hansen, J., & Nygaard, H. (2017). 10 weeks of heavy strength training improves performance-related measurements in elite cyclists. Journal of Sports Sciences, 35(14), 1435–1441. 10.1080/02640414.2016.1215499 27486014

[ref53] Sabido, R., Hernández-Davó, J. L., Botella, J., & Moya, M. (2016). Effects of 4-week training intervention with unknown loads on power output performance and throwing velocity in junior team handball players. PLoS ONE, 11(6), e0157648. 10.1371/journal.pone.0157648 27310598 PMC4911126

[ref54] Saeterbakken, A. H., Stien, N., Andersen, V., Scott, S., Cumming, K. T., Behm, D. G., Granacher, U., & Prieske, O. (2022). The effects of trunk muscle training on physical fitness and sport-specific performance in young and adult athletes: a systematic review and meta-analysis. Sports Medicine, 52(7), 1599–1622. 10.1007/s40279-021-01637-0 35061213 PMC9213339

[ref55] Saez de Villarreal, E., Suarez-Arrones, L., Requena, B., Haff, G. G., & Ramos-Veliz, R. (2014). Effects of dry-land vs. in-water specific strength training on professional male water polo players' performance. Journal of Strength & Conditioning Research, 28(11), 3179–3187. DOI: 10.1519/JSC.0000000000000514 24818541

[ref56] Saez de Villarreal, E., Suarez-Arrones, L., Requena, B., Haff, G. G., & Veliz, R. R. (2015a). Enhancing performance in professional water polo players: dryland training, in-water training, and combined training. Journal of Strength & Conditioning Research, 29(4), 1089–1097. DOI: 10.1519/JSC.0000000000000707 25259469

[ref57] Saez de Villarreal, E., Suarez-Arrones, L., Requena, B., Haff, G. G., & Ferrete, C. (2015b). Effects of plyometric and sprint training on physical and technical skill performance in adolescent soccer players. Journal of Strength & Conditioning Research, 29(7), 1894–1903. DOI: 10.1519/JSC.0000000000000838 25635606

[ref58] Saunders, P. U., Telford, R. D., Pyne, D. B., Peltola, E. M., Cunningham, R. B., Gore, C. J., & Hawley, J. A. (2006). Short-term plyometric training improves running economy in highly trained middle and long distance runners. Journal of Strength & Conditioning Research, 20(4), 947–954. 17149987 10.1519/R-18235.1

[ref59] Schärer, C., Tacchelli, L., Göpfert, B., Gross, M., Lüthy, F., Taube, W., & Hübner, K. (2019). Specific eccentric–isokinetic cluster training improves static strength elements on rings for elite gymnasts. International Journal of Environmental Research and Public Health, 16(22), 4571. 10.3390/ijerph16224571 31752246 PMC6888498

[ref60] Schoenfeld, B. J., Contreras, B., Krieger, J., Grgic, J., Delcastillo, K., Belliard, R., & Alto, A. (2019). Resistance training volume enhances muscle hypertrophy but not strength in trained men. Medicine and Science in Sports and Exercise, 51(1), 94–103. DOI: 10.1249/MSS.0000000000001764 30153194 PMC6303131

[ref61] Sedano, S., Vaeyens, R., Philippaerts, R. M., Redondo, J. C., de Benito, A. M., & Cuadrado, G. (2009). Effects of lower-limb plyometric training on body composition, explosive strength, and kicking speed in female soccer players. Journal of Strength & Conditioning Research, 23(6), 1714–1722. DOI: 10.1519/JSC.0b013e3181b3f537 19675492

[ref62] Sedano, S., Marín, P. J., Cuadrado, G., & Redondo, J. C. (2013). Concurrent training in elite male runners: the influence of strength versus muscular endurance training on performance outcomes. Journal of Strength & Conditioning Research, 27(9), 2433–2443. DOI: 10.1519/JSC.0b013e318280cc26 23287831

[ref63] Spieszny, M., Trybulski, R., Biel, P., Zając, A., & Krzysztofik, M. (2022). Post-isometric back squat performance enhancement of squat and countermovement jump. International Journal of Environmental Research and Public Health, 19(19), 12720. 10.3390/ijerph191912720 36232019 PMC9565011

[ref64] Starzak, M., Niźnikowski, T., Biegajło, M., Nogal, M., Arnista W. Ł., Mastalerz, A., Starzak, A. (2024). Attentional focus strategies in racket sports: A systematic review. PLoS ONE, 19(1), e0285239. 10.1371/journal.pone.0285239 38181000 PMC10769023

[ref65] Stone, M. H., Collins, D., Plisk, S., Haff, G., & Stone, M. E. (2000). Training Principles: Evaluation of Modes and Methods of Resistance Training. Strength & Conditioning Journal, 22(3), 65–76.

[ref66] Swann, C., Moran, A., & Piggott, D. (2015). Defining elite athletes: Issues in the study of expert performance in sport psychology. Psychology of Sport and Exercise, 16, 3–14. 10.1016/j.psychsport.2014.07.004

[ref67] Thiele, D., Prieske, O., Chaabene, H., & Granacher, U. (2020). Effects of strength training on physical fitness and sport-specific performance in recreational, sub-elite, and elite rowers: A systematic review with meta-analysis. Journal of Sports Sciences, 38(10), 1186–1195. /10.1080/02640414.2020.1745502 32216524

[ref68] Thiele, D., Prieske, O., Lesinski, M., & Granacher, U. (2020b). Effects of equal volume heavy-resistance strength training versus strength endurance training on physical fitness and sport-specific performance in young elite female rowers. Frontiers in Physiology, 11, 888. 10.3389/fphys.2020.00888 32848844 PMC7396593

[ref69] Tucker, R., & Collins, M. (2012). What makes champions? A review of the relative contribution of genes and training to sporting success. British Journal of Sports Medicine, 46(8), 555–561. 10.1136/bjsports-2011-090548 22535537

[ref70] Wang, J., Fu, H., Qiang Zhang, Zhang, M., & Fan, Y. (2022). Effect of leg half-squat training with blood flow restriction under different external loads on strength and vertical jumping performance in well-trained volleyball players. Dose-Response, 20(6), 15593258221123673. 10.1177/15593258221123673 36158741 PMC9500279

[ref71] Williams, A., Day, S., Stebbings, G., & Erskine, R. (2017). What does ‘elite’ mean in sport and why does it matter. *Sport and Exercise Scientist*, 51, 6.

[ref72] Zinke, F., Warnke, T., Gäbler, M., & Granacher, U. (2018). Effects of isokinetic training on trunk muscle fitness and body composition in world-class canoe sprinters. Frontiers in Physiology, 10, 21. 10.3389/fphys.2019.00021 PMC6360172 30745878

[ref73] Zouita, A., Darragi, M., Bousselmi, M., Sghaeir, Z., Clark, C. C., Hackney, A. C., Granacher, U., & Zouhal, H. (2023). The effects of resistance training on muscular fitness, muscle morphology, and body composition in elite female athletes: A systematic review. Sports Medicine (Auckland, N.Z.), 53(9), 1709–1735. 10.1007/s40279-023-01859-4 37289331 PMC10432341

